# Efficient Derivation of Multipotent Neural Stem/Progenitor Cells from Non-Human Primate Embryonic Stem Cells

**DOI:** 10.1371/journal.pone.0049469

**Published:** 2012-11-14

**Authors:** Hiroko Shimada, Yohei Okada, Keiji Ibata, Hayao Ebise, Shin-ichi Ota, Ikuo Tomioka, Toshihiro Nomura, Takuji Maeda, Kazuhisa Kohda, Michisuke Yuzaki, Erika Sasaki, Masaya Nakamura, Hideyuki Okano

**Affiliations:** 1 Department of Orthopedic Surgery, School of Medicine, Keio University, Tokyo, Japan; 2 Department of Physiology, School of Medicine, Keio University, Tokyo, Japan; 3 Kanrinmaru Project, School of Medicine, Keio University, Tokyo, Japan; 4 Department of Pediatrics, School of Medicine, Keio University, Tokyo, Japan; 5 Genomic Science Laboratories, Dainippon Sumitomo Pharma Co. Ltd., Osaka, Japan; 6 Marmoset Research Department, Central Institute for Experimental Animals, Kanagawa, Japan; Chiba University Center for Forensic Mental Health, Japan

## Abstract

The common marmoset (*Callithrix jacchus*) is a small New World primate that has been used as a non-human primate model for various biomedical studies. We previously demonstrated that transplantation of neural stem/progenitor cells (NS/PCs) derived from mouse and human embryonic stem cells (ESCs) and induced pluripotent stem cells (iPSCs) promote functional locomotor recovery of mouse spinal cord injury models. However, for the clinical application of such a therapeutic approach, we need to evaluate the efficacy and safety of pluripotent stem cell-derived NS/PCs not only by xenotransplantation, but also allotransplantation using non-human primate models to assess immunological rejection and tumorigenicity. In the present study, we established a culture method to efficiently derive NS/PCs as neurospheres from common marmoset ESCs. Marmoset ESC-derived neurospheres could be passaged repeatedly and showed sequential generation of neurons and astrocytes, similar to that of mouse ESC-derived NS/PCs, and gave rise to functional neurons as indicated by calcium imaging. Although marmoset ESC-derived NS/PCs could not differentiate into oligodendrocytes under default culture conditions, these cells could abundantly generate oligodendrocytes by incorporating additional signals that recapitulate *in vivo* neural development. Moreover, principal component analysis of microarray data demonstrated that marmoset ESC-derived NS/PCs acquired similar gene expression profiles to those of fetal brain-derived NS/PCs by repeated passaging. Therefore, marmoset ESC-derived NS/PCs may be useful not only for accurate evaluation by allotransplantation of NS/PCs into non-human primate models, but are also applicable to analysis of iPSCs established from transgenic disease model marmosets.

## Introduction

The common marmoset (*Callithrix jacchus*) is a small New World primate that has a relatively short gestational period (approximately 144 days), reaches early sexual maturity at 12–18 months and produces more offspring (40–80) during their life, compared with that of other experimental primate animal models such as rhesus monkeys (*Macaca mulatta*) and cynomolgus monkeys (*Macaca fascicularis*). In addition, because of its relatively small size and similar anatomical and physiological features to those of humans, marmosets have recently attracted considerable attention as a non-human primate model for biomedical research. Recently, we have successfully generated transgenic marmosets with germline transmission by lentiviral vector-mediated gene transfer [1]. Thus, our technique may provide novel non-human primate models for various biomedical studies of human diseases.

We have previously confirmed the efficacy of transplantation of rodent and human fetal neural stem/progenitor cells (NS/PCs) for functional locomotor recovery in rodent and marmoset spinal cord injury (SCI) models [2], [3]. However, due to the ethical concerns of human fetal NS/PCs, we have focused more on NS/PCs derived from pluripotent stem cells, including embryonic stem cells (ESCs) and induced pluripotent stem cells (iPSCs), considering their potential use for clinical applications. Thus far, we have found that transplantation of NS/PCs derived from mouse and human ESCs and iPSCs promotes the functional recovery of mouse SCI models [4], [5], [6]. However, for the clinical application of pluripotent stem cell-derived NS/PC transplantation, the evaluation of its efficacy and safety using rodent SCI models or xenotransplantation of human cells into rodent models are insufficient because of immunological rejection. Therefore, for a more accurate analysis, allotransplantation of non-human primate ESC/iPSC-derived NS/PCs into a non-human primate model is required.

In this study, we successfully derived NS/PCs as neurospheres from marmoset ESCs [7]. This culture system enables efficient derivation of marmoset NS/PCs that can give rise to neurons, astrocytes and oligodendrocytes. Thus, our culture system is applicable not only to derive donor cells for allotransplantation into marmoset models, but also as an *in vitro* model to analyze iPSCs derived from transgenic disease model marmosets.

## Materials and Methods

### Animals

This study was approved by the Institutional Animal Care and Use Committee of the Central Institute for Experimental Animals (CIEA), and was performed in accordance with CIEA and Keio University guidelines. Adult common marmosets (>2 y old) were purchased from a CLEA Japan (Tokyo, Japan). The animals were kept at 26°C with 65% humidity and illumination for 12 h/d. To facilitate breeding, females were paired with fertile males during the luteal phase of the menstrual cycle and were given time to adapt to their new mating partners before being entered into the experiment.

### Culture of Common Marmoset ESCs

Previously established marmoset ESCs (No. 20) [7] were cultured in common marmoset ESC (CMESC) medium consisting of Knockout Dulbecco's modified Eagle's medium (DMEM) supplemented with 20% Knockout Serum Replacement (KSR; Invitrogen, Carlsbad, CA), 1 mM L-glutamine (Nacalai Tesque, Kyoto Japan), 0.1 mM minimum essential medium (MEM) nonessential amino acids (Invitrogen), 0.1 mM 2-mercaptoethanol (2-ME; Sigma, St. Louis, MO, USA), 100 U/ml penicillin (Nacalai Tesque), 100 µg/ml streptomycin sulfate (Nacalai Tesque) and 10 ng/ml human leukemia inhibitory factor (hLIF; Millipore, Bedford, MA), on a 3,500 rad γ-irradiated mouse embryonic fibroblast (MEF) feeder layer. For passaging, undifferentiated ESC colonies were detached from feeder cells using a dissociation solution consisting of 0.25% trypsin, 1 mg/ml collagenase IV, 1 mM CaCl_2_ and 20% KSR in PBS [8], mechanically dissociated into 10–50 cell aggregates and then replated onto a fresh irradiated MEF feeder layer.

### Differentiation of ESCs

For embryoid body (EB) formation, passage 35–45 ESC colonies were detached with the dissociation solution and then plated onto ultra-low cluster culture dishes (Corning, Acton, MA, USA) in CMESC medium without hLIF after removal of MEFs by plating cells onto gelatin-coated dishes for 2 hours. On day 1, the medium was replaced with freshly prepared EB medium consisting of Knockout DMEM containing 5% KSR, 1 mM L-glutamine, 0.1 mM MEM nonessential amino acids and 0.1 mM 2- ME. For neural induction, 3 µM dorsomorphin (6-[4-(2-piperidinl-yl-ethoxy)phenyl]-3-pyridin-4-yl-pyrazolo [1,5-a] pyrimidine; Sigma) (on day 1) or 1×10^−6^ M all-trans retinoic acid (RA; Sigma, St. Louis, MO) (on day 5) were added to the culture medium. The medium was changed every 2–3 days.

For primary neurosphere formation, EBs were collected on day 14 and dissociated with TrypLE Select (Invitrogen) for 15 minutes at 37°C, followed by suspension culture at a density of 5×10^4^ cells/ml in media hormone mix (MHM) medium consisting of DMEM/F-12 (1∶1) (Gibco), 0.6% glucose, 2 mM glutamine, 3 mM sodium bicarbonate, 5 mM HEPES, 25 µg/ml insulin, 100 µg/ml transferrin, 20 nM progesterone, 30 nM selenium chloride and 60 µM putrescine (all purchased from Sigma) [9] containing 2% B27 supplement (Invitrogen) and 20 ng/ml fibroblast growth factor-2 (FGF-2) (PeproTech, Rocky Hill, NJ). The medium was changed every week and FGF-2 was added every 2 days. For secondary neurosphere formation, primary neurospheres were dissociated and cultured at a density of 5×10^4^ cells/ml in MHM medium containing 2% B27 and 20 ng/ml FGF-2. For differentiation, neurospheres were plated onto poly-L-ornithine/fibronectin-coated coverslips and allowed to differentiate without growth factors for 8–10 days.

To derive neurospheres that efficiently differentiated into oligodendrocytes, 1×10^−6^ M RA and 2 µM purmorphamine (Millipore) were added on day 5 and 7 of EB formation, respectively. Then, EBs were dissociated and cultured in suspension to form neurospheres in MHM medium containing 2% B27, 20 ng/ml FGF-2, 1 µM purmorphamine, 20 ng/ml epidermal growth factor (EGF) (Pepro Tech), 10 ng/ml platelet-derived growth factor-AA (PDGF-AA) (Pepro Tech), 10 ng/ml recombinant human neurotrophin-3 (rhNT3) (R&D, Minneapolis, MN), 10 ng/ml recombinant human insulin-like growth factor-1 (rhIGF-1) (R&D), 1 µM cyclic AMP (Sigma), 100 ng/ml biotin (Sigma) and 60 ng/ml T3 (Sigma). These neurospheres could be passaged into secondary neurospheres in the same manner described above. For differentiation into oligodendrocytes, neurospheres were plated onto poly-L-ornithine/laminin (Sigma) -coated coverslips and allowed to differentiate for 30–35 days in the presence of 10 ng/ml PDGF-AA, 10 ng/ml rhNT3, 10 ng/ml rhIGF-1, 1 µM cyclic AMP, 100 ng/ml biotin and 60 ng/ml T3.

### Caesarean Section

To obtain marmoset embryos, animals were immobilized with 30 mg/kg of ketamine hydrochloride (Veterinary Ketalar 50; Sankyo Lifetech Co., Ltd., Tokyo, Japan) and 0.075 mg/kg of atropine sulfate (Atropine Sulfate Injection; Mitsubishi Tanabe Pharma Corporation, Osaka, Japan) given by intramuscular injection. Thereafter, anesthesia was maintained by inhalation of 1.0–3.0% of isoflurane (Forane; Abbott Japan, Tokyo, Japan) via a ventilation mask. During the operation, anesthesia was managed by spontaneous respiration and heart rate and arterial oxygen saturation were monitored. The uterus was exteriorized following midline laparotomy, and the proximal end of the uterus was incised for the Caesarean section. After the Caesarean section, the uterus, abdominal muscles, and skin were sutured.

### Culture of Common Marmoset Embryonic Neurospheres

To derive neurospheres, brains and spinal cords were immediately dissected from embryonic day (E)78–91 marmoset embryos and dissociated mechanically, followed by suspension culture at a density of 2–3×10^4^ cells/ml in MHM medium containing 2% B27 supplement, 20 ng/ml FGF-2, 20 ng/ml epidermal growth factor (EGF) (PeproTech), 5 µg/ml Heparin (Sigma) and 10 ng/ml hLIF. The medium was changed every week and FGF-2 was added every 2 days.

### RNA Isolation and Reverse Transcription-Polymerase Chain Reaction (RT-PCR)

RNA isolation and RT-PCR were performed as described elsewhere [10]. Briefly, Total RNA was isolated with Trizol reagent (Invitrogen) and an RNeasy Mini Kit with DNase I treatment (Qiagen, Valencia, CA). To analyze undifferentiated ESCs, 300 ESC colonies were collected and immediately suspended in Trizol reagent. Total RNA (1–3 µg) was then used to synthesize cDNA with 500 ng oligo-d(T)_12–18_ primers. cDNA synthesis was performed at 50°C for 50 minutes in a final volume of 20 µl according to the manufacturer's instructions for Superscript III RNase H reverse transcriptase (Invitrogen). To analyze relative mRNA expression, the amount of cDNA was normalized to signals from ubiquitously expressed *β-Actin* mRNA. Quantitative (q)RT-PCR was performed using an MX3000P (Stratagene, La Jolla, CA), with SYBR Pre-mix ExTaq II (Takara, Otsu, Japan). Primer sequences and annealing temperatures are listed in Table S1. Data were expressed as the amount of mRNA relative to that of neurospheres derived from forebrain, ganglionic eminence (GE), cortex and the spinal cord of embryonic day (E)78–91 marmoset embryos, or heart, liver and spinal cord derived from E78–91 marmoset embryos.

### Calcium Imaging and Electrical Stimulation

To load the calcium imaging dye, cells were incubated with 1 µM fluo-4 AM (F23917; Invitrogen) in imaging solution consisting of 117 mM NaCl, 2.5 mM KCl, 2 mM CaCl_2_, 2 mM MgSO_4_, 25 mM HEPES and 30 mM D-(+)-glucose, (pH 7.4), at 37°C for 20 minutes, followed by washing for 30 minutes in imaging solution. Coverslips were placed on a custom-made field stimulation chamber and mounted on the stage of a Nikon Eclipse microscope with a 20× (NA 0.45) objective. Cells were perfused at 2 ml/minute with the imaging solution at room temperature with or without 1 µM tetrodotoxin (TTX; Alomone Labs Ltd. Israel). Images were acquired at 2 Hz (500 millisecond exposure time) with a cooled CCD camera (Andor iXon, DU897). Extracellular field stimulation was performed with two parallel platinum wires at 25 V/cm. Each stimulation was a train of 500 microsecond pulses at 40 Hz for 5 seconds. Images were analyzed with ImageJ software (NIH, Bethesda, MD).

### DNA Microarray Analysis

Total RNA from ESCs, EBs and neurospheres was isolated using an RNeasy Plus Micro kit (Qiagen). RNA was reverse transcribed, biotin- labeled and hybridized for 16 hours to a Marmoset Genome oligonucleotide custom array, Marmo2 [11], which was subsequently washed and stained in a Fluidics Station 450 (Affymetrix Japan, Tokyo, Japan) according to the manufacturer's instructions. Microarrays were scanned using a GeneChip Scanner 3000 7G (Affymetrix), and raw image files were converted to normalized signal intensity values using the MAS 5.0 algorithm.

### Statistical analysis

Probesets with no significant signals on Marmo2 array were excluded from following calculation. Subsequently, principal component analysis (PCA) was performed by using R software environment 2.12.1 (http://www.r-project.org) after normalizing signals to z-scores among samples. Scatter plot graph was created by using Spotfire Decision Site 9.1.2 (TIBCO Software Inc.).

### Lentivirus production and infection of neurospheres

A self-inactivating human immunodeficiency virus (HIV)-1-based lentivirus vector, pCSII-EF-Venus [12], was used to label cells for transplantation into the brains of NOD/SCID mice. For lentivirus production, HEK-293T cells were transfected with pCSII-EF-Venus [13], pCAG-HIVgp, and pCMV-VSV-G-RSV-Rev (kindly provided by Dr. H. Miyoshi, RIKEN Tsukuba Institute, Ibaraki, Japan), and the conditioned medium containing virus particles was collected. The virus was concentrated by centrifugation at 25,000 rpm for 1.5 h at 4°C. The concentrated virus particles were added to the culture medium, in which secondary neurospheres were then generated from primary neurospheres. Infected Secondary neurospheres were used for transplantation into the brains of NOD/SCID mice.

### Transplantation of marmoset ESC-derived neurospheres

For transplantation, Venus-labeled secondary neurospheres were partially dissociated and resuspended at a density of approximately 1.0×10^5^ cells/µl. 6–8-week-old female NOD/SCID mice (Charles River Inc.) were deeply anesthetized and cell suspension was transplanted into the right striatum of the brain by using a glass micropipette fitted to a stereotaxic injector (Narishige), as previously described [14]. The tip of the micropipette was inserted into the right striatum (2 mm lateral, 1 mm rostral to bregma; depth, 3 mm from dura), and a cell suspensions of secondary neurospheres (5 µl; 5×10^5^ cells) were injected. At 4 weeks after injection, animals were perfused, and brains were removed and processed for histological analysis.

### Histological Analysis

Differentiated neurospheres were fixed with 4% paraformaldehyde for 20 minutes at room temperature and then processed for immunocytochemistry. Samples were rinsed with PBS twice and blocked in PBS containing 10% fetal bovine serum and 0.3% Triton X-100 for 1 hour at room temperature. Then, samples were incubated at 4°C overnight with the following antibodies: anti-βIII tubulin (mouse IgG_2b_, 1∶500; Sigma), Alexa488-conjugated anti-βIII tubulin (mouse IgG, 1∶2,000, Covance, Princeton NJ), anti-glial fibrillary acidic protein (GFAP) (rabbit IgG, 1∶10,000, Dako, Glostrup, Denmark), anti-2′, 3′-cyclic nucleotide 3′-phosphodiesterase (CNPase) (mouse IgG_1_, 1∶4,000, Sigma), anti-human Nestin (rabbit IgG, 1∶4,000) [15], anti-myelin basic protein (MBP) (rat IgG, 1∶500, AbD Serotec, Raleigh, NC, USA), anti-O4 (mouse IgM 1∶1,000, Millipore), and anti-platelet-derived growth factor receptor (PDGFR)α (rabbit IgG, 1∶2,000, Santa Cruz, Santa Cruz, CA, USA).

After three washes with PBS, samples were incubated for 1 hour at room temperature with secondary antibodies conjugated with Alexa488, Alexa555 or Alexa647 (Invitrogen). Nuclei were stained with Hoechst 33258 (Sigma). After washing with PBS, samples were mounted on slides and examined under a universal fluorescence microscope (Axiophot 2; Carl Zeiss).

For the immunohistochemical (IHC) analysis, transplanted mice were anesthetized and transcardially perfused with 4% PFA and post-fixed overnight. 40 µm sections were prepared from the brains with a vibrating blade microtome (VT1000S; Leica) for IHC analysis. After a 1 hour incubation at room temperature in blocking buffer (TNB buffer (PerkinElmer, Waltham, MA, USA) containing 0.3% Triton-X 100, or PBS containing 10% normal goat serum and 0.3% Triton-X 100), the samples were incubated with primary antibodies : anti-GFP (rabbit IgG, 1∶500; MBL, Aichi, Japan), anti-GFP (goat IgG, 1∶1,000; Rockland, Gilbertsville, PA, USA), anti-Hu (Human serum, 1∶5000; Gift from Dr. Robert B. Darnell), anti-GFAP (rat IgG, 1∶500; Zymed, South San Francisco, CA, USA), anti-APC CC-1 (mouse IgG, 1∶200; Calbiochem, San Diego, CA, USA), and anti-human Nestin (rabbit IgG, 1∶4,000) [15] at 4°C overnight. After washing three times with PBS, the samples were incubated with secondary antibodies conjugated with Alexa488, Alexa555, or Alexa647 (Invitrogen) for 2 hours at room temperature. The nuclei were stained with Hoechst33258. The samples were examined with a confocal laser scanning microscope (LSM700, Carl Zeiss).

## Results

### Derivation of Neurospheres from Marmoset ESCs

**Figure 1:**
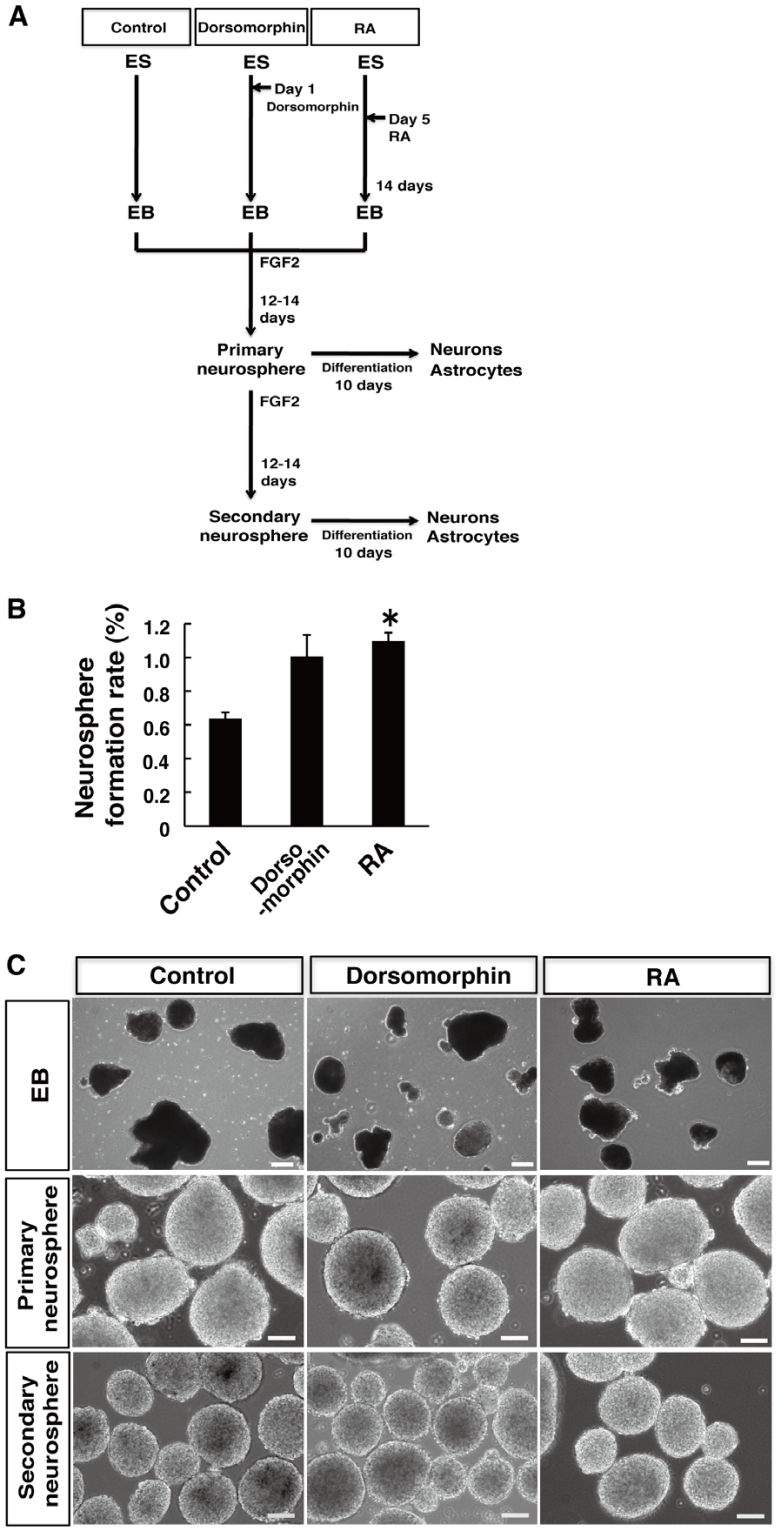
Neurosphere formation of marmoset ESCs. (**A**) Protocol to derive neurospheres from marmoset ESCs by EB formation. EBs were cultured in suspension in ultra-low cluster dishes for 2 weeks in the presence of 3 µM dorsomorphin or 1×10^−6^ M RA. Dorsomorphin or RA were added on day 1 or 5 of EB formation, respectively. EBs were then dissociated and cultured in suspension for 12–14 days to form neurospheres in MHM medium containing 2% B27 and 20 ng/ml FGF-2. Primary neurospheres were dissociated and cultured in suspension again with FGF-2 to form secondary neurospheres. (**B**) Neurosphere formation rates are presented as the percentages of neurospheres among total cells plated. EBs treated with 3 µM dorsomorphin or 1×10^−6^ M RA were dissociated and cultured in MHM medium containing 2% B27 and 20 ng/ml FGF-2 at a density of 2.5×10^4^ cells/ml in an ultra-low cluster 96-well plate for 1 week, and then neurospheres larger than 50 µm in diameter were counted. Data are presented as the means ± SEM (*n* = 3). (**C**) Representative morphologies of EBs, primary neurospheres and secondary neurospheres under each condition. Scale bars, 100 µm for EBs, 200 µm for neurospheres.

By modifying our own methods for neural differentiation of mouse and human ESCs and iPSCs [16], we derived NS/PCs as neurospheres through EB formation from marmoset ESCs (No. 20) (Fig. 1A). Although we added noggin on day 0 or RA on day 2 during 6 days of EB formation to differentiate mouse ESCs into NS/PCs, for marmoset ESC differentiation, we cultured EBs in suspension for 2 weeks, because the gestational period of marmosets is longer than that of mice. We also added 3 µM dorsomorphin, a selective chemical inhibitor of bone morphogenetic protein (BMP) signaling, instead of noggin [17] on day 1 or 1×10^−6^ M RA on day 5. EBs were then dissociated and cultured in suspension for 12–14 days to form neurospheres in serum-free MHM medium containing FGF-2. As a result, administration of 3 µM dorsomorphin or 1×10^−6^ M RA during EB formation increased the efficiency of neurosphere formation (1.01±0.13% and 1.10±0.05% in neurospheres derived from dorsomorphin-treated EBs [D-NS] and RA-treated EBs [RA-NS], respectively) compared with that of the control (0.64±0.04% in neurospheres derived from control EBs [C-NS]). (Fig. 1B), suggesting that dorsomorphin and RA promoted neural differentiation of marmoset ESCs, as was observed with mouse ESCs. Moreover, these marmoset ESC-derived primary neurospheres could be dissociated and cultured again in suspension to form secondary neurospheres, and passaged similarly to at least tertiary neurospheres, indicating that they had the ability to self-renew (Fig. 1C).

### Primary and Secondary Neurospheres Sequentially Acquire Neurogenic and Gliogenic Potentials, Respectively

**Figure 2:**
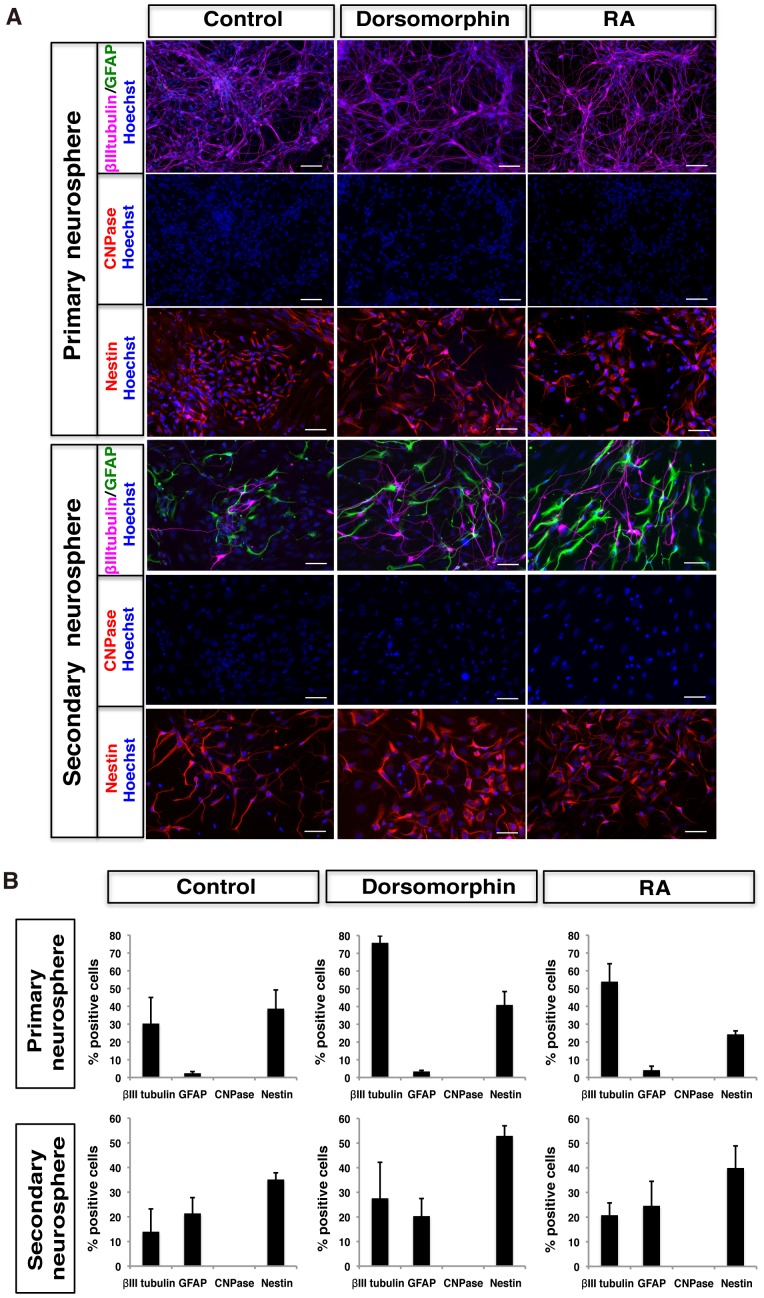
Differentiation potentials of marmoset ESC-derived NS/PCs. (**A**) Marmoset ESC-derived primary and secondary neurospheres were dissociated and allowed to differentiate for 10 days, followed by immunocytochemical analysis of βIII-tubulin (neurons), GFAP (astrocytes), CNPase (oligodendrocytes) and Nestin (undifferentiated neural cells). Scale bars, 50 µm. (**B**) The proportions of cells positive for each cell type-specific marker are presented as the percentage of total cells counted by Hoechst 33258-stained nuclei. Data are presented as the means ± SEM (*n* = 3).

We next examined the differentiation potentials of marmoset ESC-derived neurospheres. Neurospheres were dissociated and differentiated adherently for 10 days without FGF-2, followed by immunocytochemical analysis of markers for neurons (βIII tubulin), astrocytes (GFAP), and oligodendrocytes (CNPase) (Fig. 2A, B). Most cells derived from primary neurospheres differentiated into βIII tubulin-positive neurons (75.9±3.9% in D-NS, 53.9±10.0% in RA-NS, and 30.4±14.7% in C-NS), whereas only a small number of cells differentiated into glial cells (<5%). In contrast, when secondary neurospheres were differentiated with the same method, they generated both βIII tubulin-positive neurons and GFAP-positive astrocytes (27.6±14.6% neurons and 20.3±7.1% astrocytes in D-NS, 20.8±5.0% neurons and 24.6±9.9% astrocytes in RA-NS, and 14.0±9.2% neurons and 21.4±6.4% astrocytes in C-NS). However, CNPase-positive oligodendrocytes could not be detected under this condition. Nestin-positive undifferentiated cells were also observed in differentiated primary neurospheres (40.9±7.6% in D-NS, 24.2±2.0% in RA-NS and 38.7±10.5% in C-NS), and in differentiated secondary neurospheres (52.9±2.4% in D-NS, 40.0±9.0% in RA-NS and 35.1±2.7% in C-NS). These results indicated that marmoset ESC-derived neurospheres could differentiate into neurons and astrocytes, but not oligodendrocytes under any of these conditions. Interestingly, the sequential acquisition of neurogenic and gliogenic potentials in primary and secondary neurospheres is consistent with *in vivo* central nervous system (CNS) development in mice, in which neurons are generated first and glial cells later [16].

**Figure 3:**
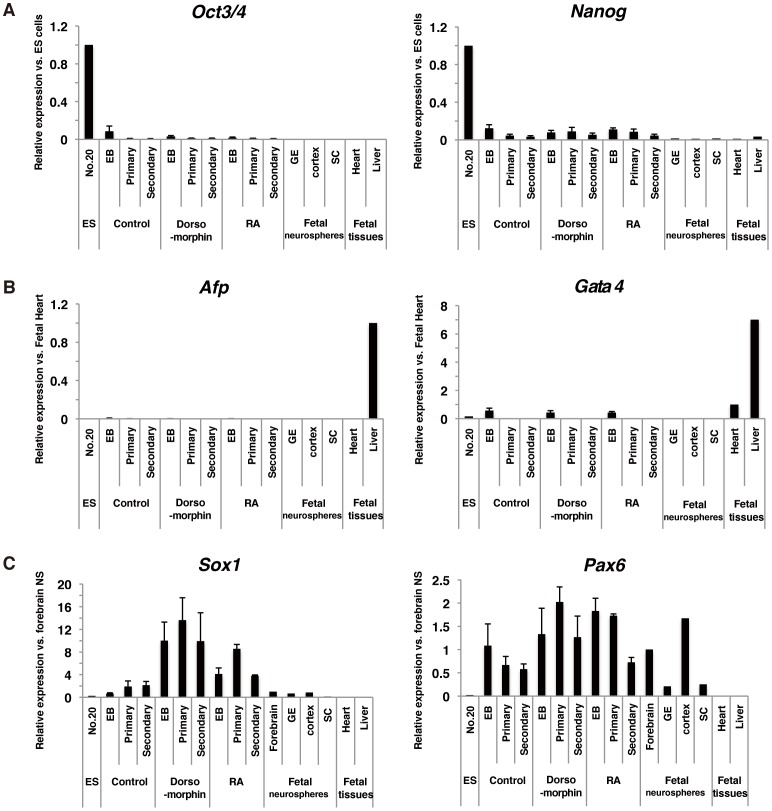
Expression of cell type-specific markers at each differentiation step. qRT-PCR analysis of the expression of pluripotency markers (A: *Oct3/4* and *Nanog*), endodermal and mesodermal markers (B: *Afp* and *Gata4*, respectively), and neural markers (C: *Sox1* and *Pax6*) in ESCs, EBs and ESC-derived neurospheres. Fetal cell-derived (ganglionic eminence (GE), cortex and spinal cord) neurospheres and fetal tissues (heart and liver) were used as positive and negative controls, respectively. Data are presented as the means ± SEM (*n* = 3).

To further evaluate the characteristics of these neurospheres, we examined the expression of cell type-specific markers in EBs, as well as primary and secondary neurospheres. Pluripotency markers, *Oct3/4* and *Nanog*, were immediately downregulated by neurosphere formation (Fig. 3A). An endodermal marker, *Afp*, and a mesodermal marker, *Gata4*, were slightly expressed in EBs, but significantly downregulated in neurospheres (*p<0.05* vs. ESCs) (Fig. 3B), suggesting that neurosphere formation in serum-free medium promoted the differentiation of neural cells by selection of NS/PCs in culture. In contrast, the expression of *Sox1* and *Pax6* were significantly upregulated by neural differentiation of ESCs (Fig. 3C). Moreover, the expression of *Sox1* in dorsomorphin- and RA-treated EBs was 15.1- and 6.2-fold higher, respectively, than those of control EBs, suggesting that neural differentiation of marmoset ESCs was efficiently promoted by this method.

### Rostrocaudal Identities of Marmoset ESC-derived Neurospheres

**Figure 4:**
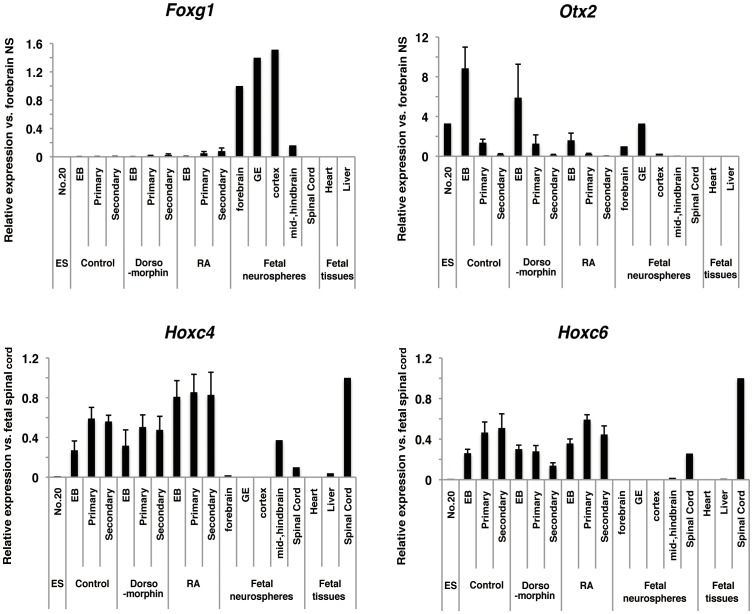
Rostrocaudal regulation of marmoset ESC-derived NS/PCs. Rostrocaudal regional identities of marmoset ESC-derived neurospheres. qRT-PCR analysis of the expression of forebrain marker *Foxg1*, midbrain marker *Otx2* and spinal cord markers *Hoxc4* and *Hoxc6* in ESCs, EBs and ESC-derived neurospheres. Fetal cell-derived (ganglionic eminence (GE), cortex and spinal cord) neurospheres and fetal tissues (heart and liver) were used as positive and negative controls, respectively. Data are presented as the means ± SEM (*n* = 3).

Because noggin and RA are involved in regulation of rostrocaudal identities during *in vivo* neural development, we further examined whether we could observe similar rostrocaudal regulation in marmoset ESC-derived NS/PCs, compared with that observed in mouse ESC-derived NS/PCs [16]. We found that D-NS did not express the forebrain marker *Foxg1*, but did express forebrain-to-midbrain marker *Otx2* at the same level as that in embryonic forebrain neurospheres (Fig. 4). The expression of caudal hindbrain to rostral spinal cord markers, *Hoxc4* and *Hoxc6*, were higher in D-NS, (5- and 1-fold higher, respectively, than those in fetal spinal cord neurospheres). However, RA-NS did not express *Foxg1* and *Otx2*, but did express spinal cord markers *Hoxc4* and *Hoxc6* (8- and 2.5-fold higher, respectively, than those in fetal spinal cord neurospheres) (Fig. 4). These results suggested that D-NS had a midbrain-rostral spinal cord regional identity, and RA-NS had a rostral-spinal cord regional identity. In contrast to clear rostrocaudal regulation by noggin and RA in mouse ESC-derived NS/PCs, the rostrocaudal identities of marmoset ESC-derived NS/PCs were not as clearly regulated by dorsomorphin and RA, although RA slightly caudalized NS/PCs.

### Differentiation into Oligodendrocytes

**Figure 5:**
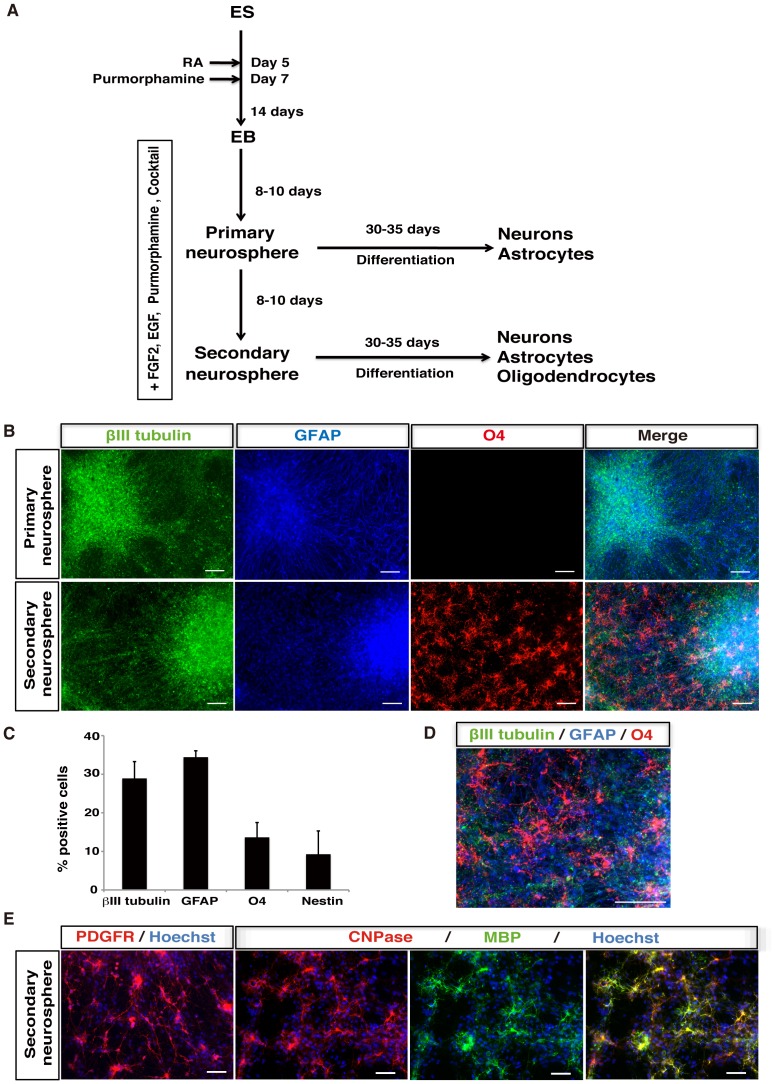
Efficient derivation of oligodendrocytes from marmoset ESCs. (**A**) Protocol for derivation of NS/PCs from marmoset ESCs, which efficiently generate oligodendrocytes. EBs were cultured in the presence of 1×10^−6^ M RA and 2 µM purmorphamine for 2 weeks. RA and purmorphamine were added on day 5 and 7 of EB formation, respectively. EBs were then dissociated and cultured in suspension for 8–10 days to form neurospheres in MHM medium containing 2% B27, 20 ng/ml FGF-2, 20 ng/ml EGF, 1 µM purmorphamine and an oligodendrocyte cocktail. Primary neurospheres were dissociated and cultured under the same condition to form secondary neurospheres. (**B**) Differentiation of neurospheres derived from EBs treated with RA and purmorphamine. Primary and secondary neurospheres were dissociated and differentiated on poly-L-ornithine/laminin-coated coverslips at a density of 2×10^4^ cells/ml in MHM medium containing 2% B27 and an oligodendrocyte cocktail for 30–35 days, followed by immunocytochemical analysis of βIII-tubulin (neurons), GFAP (astrocytes) and O4 (oligodendrocytes). The obtained NS/PCs could efficiently give rise to oligodendrocytes. Scale bars, 100 µm. (**C**) The proportions of cells positive for each cell type-specific marker in differentiated secondary neurospheres, which could generate oligodendrocytes, are presented as the percentage of total cells counted by Hoechst 33258-stained nuclei. Data are presented as the means ± SEM (*n* = 3). (**D**) An enlarged image of βIII-tubulin-positive neurons, GFAP-positive astrocytes and O4-positive oligodendrocytes differentiated from secondary neurospheres. Scale bars, 100 µm. (**E**) Immunocytochemical analysis of secondary neurospheres for various markers of oligodendrocyte lineages, including PDGFR (OPCs), CNPase (oligodendrocytes) and MBP (mature oligodendrocytes). Scale bars, 50 µm.

Although we could efficiently generate neurons and astrocytes from marmoset ESC-derived neurospheres, we could not detect CNPase-positive oligodendrocytes under any culture condition (Fig. 2). Therefore, we explored the generation of oligodendrocytes by modifying the default culture conditions (Fig. 5A). Because purmorphamine, a small molecule that activates the hedgehog signaling pathway, promotes ventralization, and EGF is required for expansion of NS/PCs with the potential to generate oligodendrocytes [18], we added 1×10^−6^ M RA on day 5, and subsequently added 2 µM purmorphamine on day 7 during EB formation for neurosphere formation in serum-free MHM medium in the presence of not only FGF-2, but also EGF. Moreover, these neurospheres were cultured in the presence of purmorphamine, T3, PDGF-AA, IGF-1, NT3, cyclic AMP and biotin (collectively designated as an oligodendrocyte cocktail), which are reported to promote oligodendrocyte differentiation [19]. Under this condition, neurospheres were efficiently formed and could be passaged repeatedly. Primary and secondary neurospheres were allowed to differentiate on poly-L-ornithine/laminin-coated coverslips for 30–35 days in serum-free MHM medium containing the same factors used for neurosphere formation, except for FGF-2 and EGF, followed by immunocytochemical analysis of βIII tubulin (neurons), GFAP (astrocytes) and O4 (oligodendrocytes) (Fig. 5B). As a result, primary neurospheres generated βIII tubulin-positive neurons and GFAP-positive astrocytes, whereas secondary neurospheres could give rise to not only βIII tubulin-positive neurons (28.9±4.4%) and GFAP-positive astrocytes (34.4±1.7%), but also O4-positive oligodendrocytes (13.7±3.8%) (Fig. 5C). We also examined the expression of PDGFRα, a marker of oligodendrocyte precursor cells (OPCs), and markers of oligodendrocytes (CNPase and MBP), and found that neurospheres could differentiate into PDGFRα-positive OPCs as well as CNPase- and MBP-positive mature oligodendrocytes (Fig. 5D). Thus, by recapitulating *in vivo* nervous system development, we could successfully differentiate marmoset ESCs into oligodendrocytes by neurosphere formation.

### Functional Properties of Neurons Generated from Marmoset ESC-derived Neurospheres

**Figure 6:**
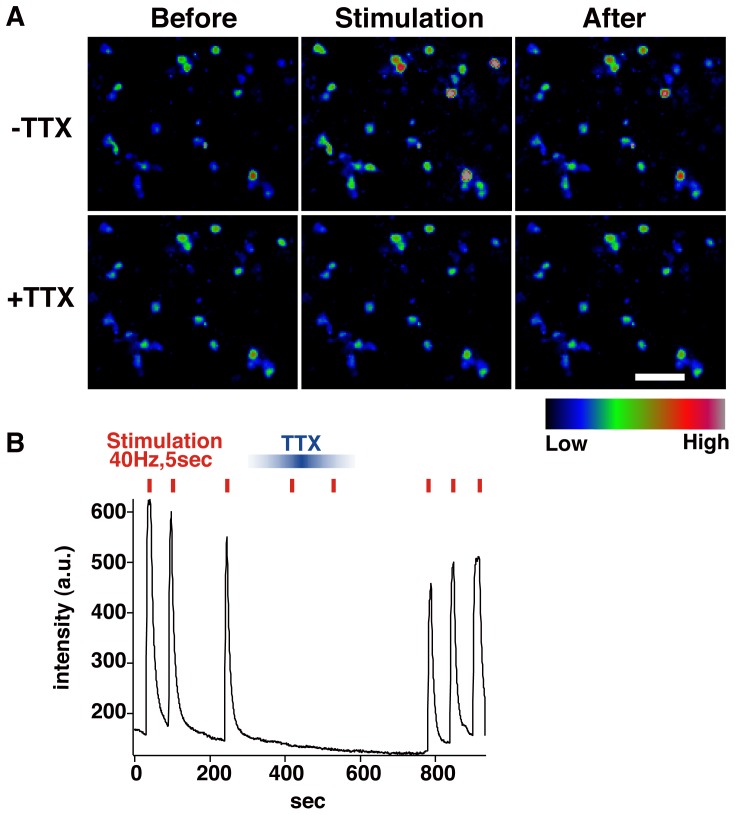
Marmoset ESC-derived NS/PCs give rise to functional neurons. NS/PCs that could generate oligodendrocytes were allowed to differentiate for 10–11 weeks. Then, [Ca^2+^]_i_ in differentiated cells were measured by the intensity of fluo-4 fluorescence upon electrical field stimulation at 40 Hz for 5 seconds. Electrical field stimulation induced an elevation of [Ca^2+^]_i_ in several cells (**A**). The intensity of the increase of [Ca^2+^]_i_ was quantified (**B**). The increase of [Ca^2+^]_i_ was suppressed by TTX that specifically blocks voltage-gated sodium channels. Scale bars, 50 µm.

To test whether the neurons generated by our *in vitro* system were functional, NS/PCs derived from EBs treated with both RA and purmorphamine, which could efficiently generate oligodendrocytes, were allowed to differentiate for 10–11 weeks. Then, intracellular calcium concentrations ([Ca^2+^]_i_) in differentiated cells were measured by the intensity of fluo-4 fluorescence in electrical field stimulation at 40 Hz for 5 seconds. As shown in Figure 6, [Ca^2+^]_i_ was dramatically increased in several cells by electrical field stimulation (Fig. 6, Movie S1). Moreover, in the presence of TTX, which specifically blocks voltage-gated sodium channels, the increase of [Ca^2+^]_i_ upon electrical field stimulation was abrogated, suggesting that the [Ca^2+^]_i_ increase was likely evoked by action potentials through voltage gated sodium channels. These results suggested that marmoset ESC-derived neurospheres could generate functional neurons.

### Marmoset ESC-derived Neurospheres Acquire Similar Properties to those Observed in Marmoset Embryonic Neurospheres

**Figure 7:**
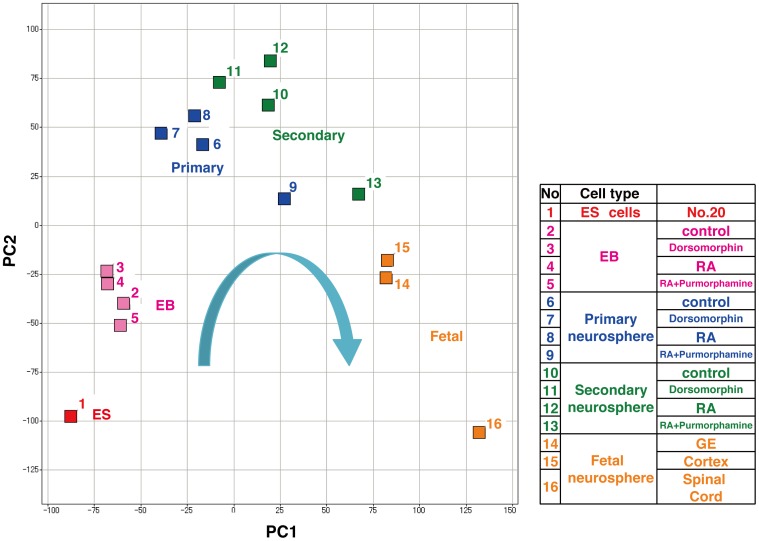
Marmoset ESC-derived neurospheres acquire similar properties to those observed in marmoset embryonic neurospheres. PCA of all microarray data was conducted on cells at each differentiation step. Undifferentiated ESCs acquired gene expression profiles close to those of fetal neurospheres after passaging into secondary neurospheres.

We performed global gene expression analyses at each step of neural differentiation to investigate whether marmoset ESC-derived neurospheres acquired properties similar to those of marmoset embryonic neurospheres, and how marmoset ESCs acquired the properties of neural progenitor cells by principal component analysis (PCA) using all microarray data (Fig. 7). Although EBs already express neural markers such as Sox1, they still have an immature characteristic and resemble undifferentiated ESCs to some extent in terms of gene expression profiles. However, ESC-derived NS/PCs transitioned from neurogenic to gliogenic NS/PCs, by passaging primary neurospheres to secondary neurospheres, and acquired expression profiles close to those of fetal brain-derived NS/PCs in secondary neurospheres. Interestingly, primary and secondary neurospheres, which could generate oligodendrocytes, exhibited gene expression profiles resembling embryonic neurospheres and particularly those derived from the GE and cortex. These results indicated that this *in vitro* marmoset ESC differentiation system may recapitulate *in vivo* marmoset neural development.

### Marmoset ESC-derived Neurospheres Survived and Differentiated into Neurons, Astrocytes and Oligodendocytes in Mouse Brain

**Figure 8:**
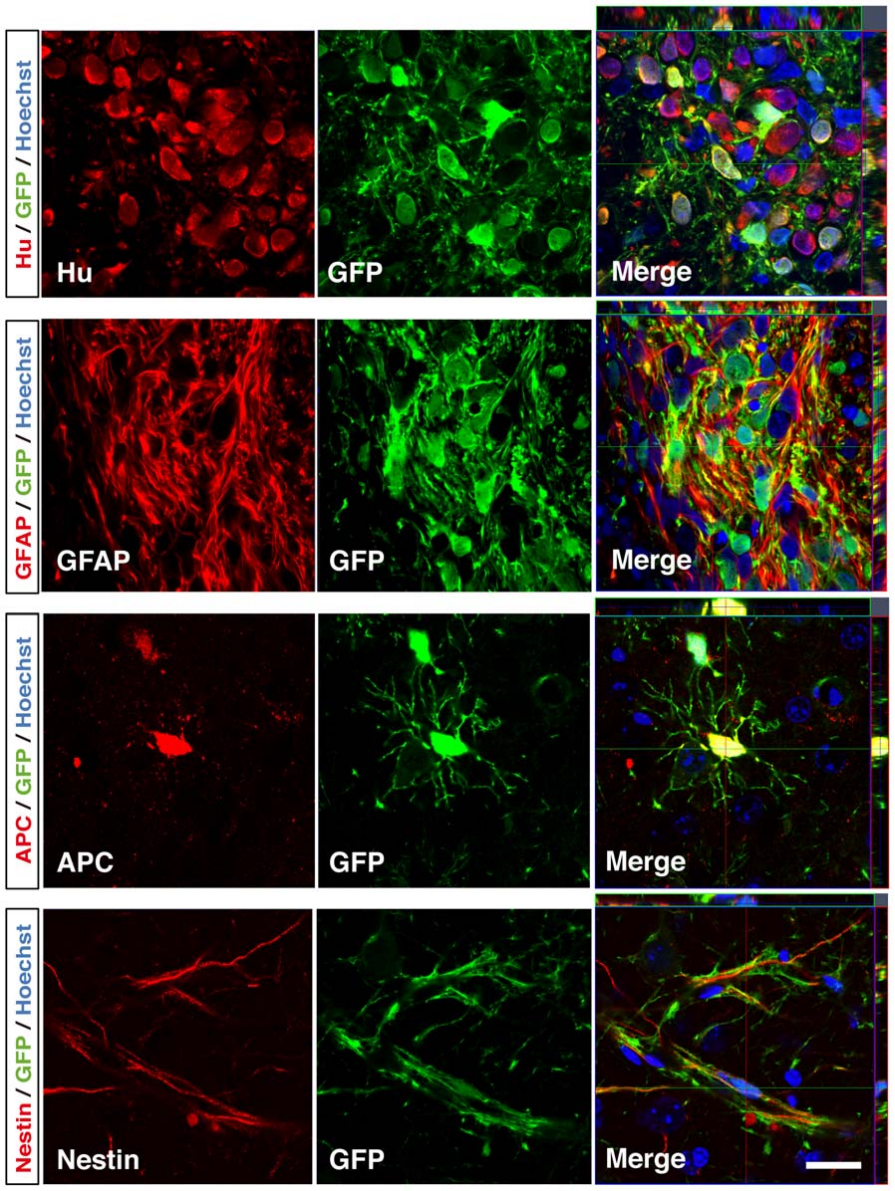
Transplantation of marmoset ESC-derived neurospheres. NS/PCs derived from EBs treated with both RA and purmorphamine, were transplanted into the right striatum of 6–8-week-old female NOD/SCID mice. 4 weeks after the injection, mice were sacrificed and processed for immunohistochemical analysis with the antibodies against markers for neurons (Hu), astrocytes (GFAP), oligodendrocytes (APC), and neural progenitors (Nestin). The marmoset ESC-derived NS/PCs could give rise to neurons, astrocytes and oligodendrocytes *in vivo*, as well. Scale bars, 20 µm.

Finally, we transplanted NS/PCs derived from EBs treated with both RA and purmorphamine, into the right striatum of 6–8-week-old female NOD/SCID mice to assess the *in vivo* differentiation potentials of the marmoset ESC-derived NS/PCs. 4 weeks later, mice were sacrificed and processed for immunohistochemical analysis. The GFP-positive transplanted ESC-derived NS/PCs survived in the striatum as cell clusters and differentiated into Hu-positive neurons, GFAP-positive astrocytes and APC-positive oligodendrocytes (Fig. 8). A small numbers of Nestin-positive neural progenitors were also observed. Thus, our marmoset ESC-derived NS/PCs could also differentiate into three types of neural cells *in vivo*.

## Discussion

In this study, we established a system for marmoset ESC differentiation into NS/PCs as neurospheres. Marmoset ESCs could efficiently form neurospheres that could be passaged repeatedly and gave rise to neurons, astrocytes and oligodendrocytes. Interestingly, neurospheres initially showed a neurogenic potential at the primary stage, and subsequently acquired gliogenic potentials in secondary neurospheres (Fig. 2). This sequential cytogenesis recapitulated *in vivo* central nervous system development in mice, as is the case with neural differentiation of mouse ESCs [16]. Thus, our culture system could be used as an *in vitro* model of marmoset CNS development.

We found that small molecules, dorsomorphin and RA, promoted neurosphere formation of marmoset ESCs (Fig. 1B). Although mouse ESCs are differentiated into NS/PCs by noggin or RA, we used dorsomorphin instead of noggin for marmoset ESC differentiation. Dorsomorphin inhibits BMP signals by selective inhibition of BMP type 1 receptors ALK2, ALK3 and ALK6, resulting in the reduction of SMAD1/5/8 phosphorylation [20], thus, recapitulating the action of the BMP receptor antagonist noggin. RA is a neural differentiation inducer of ESCs and a caudalization factor of neural progenitors [10]. Both dorsomorphin and RA promoted neural differentiation of marmoset ESCs. However, rostrocaudal identities of marmoset ESC-derived NS/PCs were not so clearly regulated by dorsomorphin and RA, compared with those of mouse ESC-derived NS/PCs [10], [16]. Because even D-NS showed relatively caudal identities by expressing *Hoxc4* without RA treatment, the species differences between marmosets and mice, the status of marmoset ESCs (e.g. naïve or primed) and components in the culture medium may have affected rostrocaudal regulation in marmoset ESC-derived NS/PCs.

In mouse ESCs, neurospheres derived from noggin- and RA-treated EBs can give rise to oligodendrocytes [16]. Moreover, effective derivation of OPCs and oligodendrocytes from mouse ESCs has been achieved using RA, PDGF-AA, T3 and so on [21], [22]. In contrast, many researchers have attempted human ESC differentiation into OPCs or oligodendrocytes over the past decade, but there are only a few recent reports indicating the successful differentiation of human ESCs and iPSCs into oligodendrocytes using the medium containing RA, Sonic hedgehog (Shh), PDGF-AA and T3 [19], [23], [24]. As for non-human primate ESCs, rhesus monkey ESCs have been successfully differentiated into neural cells by EB formation and neural rosettes, but this method is inefficient to obtain oligodendrocytes [25]. Cynomolgus monkey ESCs have been reported to differentiate into various neural subtypes [12], [26], but have never been reported to differentiate into oligodendrocytes. In the current study, we could not obtain oligodendrocytes using the default culture protocol using only dorsomorphin or RA. However, we successfully developed a simple culture method to efficiently obtain oligodendrocytes by incorporating purmorphamine, as a ventralizing factor, into the protocol using RA-NS and other signaling molecules that recapitulate *in vivo* neural development (Fig. 5). If this principle is applicable to human ESCs and iPSCs, it would be useful for various studies of regenerative medicine and pathophysiological analysis of SCI and dysmyelinating disorders.

Moreover, PCA of microarray data showed that, although marmoset ESC-derived NS/PCs had distinct profiles from those of fetal brain-derived NS/PCs at first, they transitioned from neurogenic to gliogenic NS/PCs and acquired gene expression profiles close to those of fetal brain-derived NS/PCs in secondary neurospheres. Importantly, NS/PCs derived from EBs treated with both RA and purmorphamine, which could generate oligodendrocytes efficiently, showed more similar expression profiles to those of fetal NS/PCs and particularly those derived from the GE and cortex, and distinct expression profiles from those of other ESC-derived NS/PCs that could not generate oligodendrocytes. These results indicate that the Shh signaling pathway, activated by purmorphamine, promoted *in vivo* marmoset neural development in this system. In addition, this observation is consistent with the fact that fetal spinal cord-derived NS/PCs, which generate more oligodendrocytes, show different expression profiles from those derived from fetal forebrains (GE and cortex). Taken together, our culture system recapitulates *in vivo* CNS development *in vitro*, and provides a valuable *in vitro* model that may contribute to elucidation of the molecular mechanisms underlying neural development in non-human primates, which is difficult to study *in vivo*.

We have previously reported functional recovery after transplantation of rodent or human NS/PCs into the injured spinal cords of rodents and marmosets [2], [3]. Furthermore, recent studies show that transplantation of NS/PCs derived from mouse ESCs and iPSCs, as well as human iPSCs into mouse SCI models is effective for motor function recovery [4], [5], [6]. These studies have shown the roles of graft-derived neurons in circuitry reconstruction including synapse formation [6], [27], and have emphasized the crucial roles of glial cells [5], [28]. For example, we demonstrated that transplantation of mouse ESC-derived NS/PCs, which can give rise to both neurons and glial cells, into the injured spinal cords of mice is more effective than that of NS/PCs that can only generate neurons [4]. This indicates that not only neuronal regeneration but also trophic support by astrocytes and remyelination by oligodendrocytes may play significant roles in cell transplantation therapies. Because marmoset ESC-derived NS/PCs could give rise to neurons, astrocytes and oligodendrocytes both *in vitro* and *in vivo* (Figs. 2, 5 and 8), they may be a promising cell source for transplantation therapies for synapse formation, neurotrophic support and remyelination of the injured spinal cord.

Another important issue is the need for strict evaluation of the efficacy and safety of cell transplantation therapies. Although rodent models have been commonly used for analysis of SCI, rodents have marked differences in the organization of motor systems and behaviors from those of primates, and thus have limitations as animal models. For example, the corticospinal tract, which plays the most important roles in locomotor functions, is located in the posterior funiculus in rodents, but a major portion of this structure is present in the lateral funiculus with the remaining in the anterior funiculus in primates such as humans and monkeys [29], [30]. Moreover, unlike rodents, interruption of cortical projections to the spinal cord causes a major impairment in fine motor function of the hands and feet in primates [31], [32]. In addition, inflammatory and immune responses are different between rodents and primates, and such immunogenicity limits the xenografting of human cells into mouse or non-human primate models for appropriate evaluations. Thus, pre-clinical evaluation by allotransplantation of non-human primate ESC-derived NS/PCs into a non-human primate model would be necessary before human clinical trials [31], [33].

Finally, our culture system for derivation of NS/PCs from marmoset ESCs would be applicable to marmoset iPSCs that may be established from disease model marmosets [1], [11]. Thus, it would be beneficial not only for preclinical studies of cell therapies, but also *in vitro* phenotypic analysis of marmoset disease models and various applications using marmoset models and iPSCs.

## Conclusions

In the present study, we established an *in vitro* culture method to efficiently derive multipotent NS/PCs from common marmoset ESCs. These marmoset ESC-derived neurospheres can be passaged repeatedly and sequentially give rise to neurons and glial cells including both astrocytes and oligodendrocytes. Importantly, marmoset ESC-derived NS/PCs could generate functional neurons *in vitro*, and differentiated into neurons and glial cells *in vivo*, as well. They showed similar expression profiles to those of fetal brain-derived neurospheres by repeated passaging. Marmoset ESC-derived NS/PCs are applicable to pre-clinical studies of cell replacement therapies using non-human primate models, and also various studies using iPSCs established from marmoset disease models.

## Supporting Information

Table S1 **The primer sets we used in this study.**
(DOC)

Movie S1 **Marmoset ESC-derived NS/PCs give rise to functional neurons.** NS/PCs that could generate oligodendrocytes were allowed to differentiate for 10–11 weeks. Then, [Ca^2+^]_i_ in differentiated cells were measured by the intensity of fluo-4 fluorescence upon electrical field stimulation at 40 Hz for 5 seconds. Electrical field stimulation induced an elevation of [Ca^2+^]_i_ in several cells.
(WMV)
