# Supplementary material for: Efficient Derivation of Multipotent Neural Stem/Progenitor Cells from Non-Human Primate Embryonic Stem Cells
Source: PLoS One. 2012 Nov 14;7(11):e49469. doi: 10.1371/journal.pone.0049469 (PMC3498141; doi:10.1371/journal.pone.0049469)
Supplement: Table S1 — The primer sets we used in this study. (DOC) [file pone.0049469.s001.doc]

| Table S1 | |  |  |  |
| --- | --- | --- | --- | --- |
| Gene | Primer sequences (5’–3’) | | Annealing temp (ºC) |  |
|  | Forward | Reverse |  |  |
| *Sox1* | CACAACTCGGAGATCAGCAA | GTCCTTCTTGAGCAGCGTCT | 62 |  |
| *Pax6* | TGTCCAACGGATGTGTGAGT | TTTCCCAAGCAAAGATGGAC | 62 |  |
| *GATA4* | CTCCAGCAGTGCCACCAC | CTGGTCTGCGGAGACTGG | 68 |  |
| *AFP* | CCTTGTGAAGCAAAAGCCAC | CTCCCAAAGCAGCACGAC | 62 |  |
| *Oct3/4* | AACACGGAGGAGTCCCAAGA | CCCAGGGTGATCCTCTTCTG | 62 |  |
| *Nanog* | GCCTGGAGCAGTCCCTTCTA | TCCAAGTCACTGGCAGGAGA | 62 |  |
| *Foxg1* | TCTTTGCCAAGTTTTACGACG | TCGCTGACACTCCACACCT | 62 |  |
| *Otx2* | CCATGGGTACCAATGCAGTC | CCTGGAATTTCCACGAGGAT | 62 |  |
| *Hoxc4* | TCCTCTCACCTTGTCCCTTG | AATTCACCCAAACCAGACCA | 62 |  |
| *Hoxc6* | CCTATGGAGCGGCCGTT | TCCAGGGTCTGGTACCGG | 62 |  |
| *β-actin* | GGCATCCACGAAACTACCTTT | ACACTGAGTACTTGCGCTCG | 62 |  |
